# The Oral Microbiome of Denture Wearers Is Influenced by Levels of Natural Dentition

**DOI:** 10.1371/journal.pone.0137717

**Published:** 2015-09-14

**Authors:** Lindsay E. O’Donnell, Douglas Robertson, Christopher J. Nile, Laura J. Cross, Marcello Riggio, Andrea Sherriff, David Bradshaw, Margaret Lambert, Jennifer Malcolm, Mark J. Buijs, Egija Zaura, Wim Crielaard, Bernd W. Brandt, Gordon Ramage

**Affiliations:** 1 Glasgow Dental School, School of Medicine, College of Medicine, Veterinary and Life Sciences, University of Glasgow, 378 Sauchiehall Street, Glasgow, G2 3JZ, United Kingdom; 2 GlaxoSmithKline, St Georges Avenue, Weybridge, Surrey, United Kingdom; 3 Department of Preventive Dentistry, Academic Centre for Dentistry Amsterdam, University of Amsterdam and VU University Amsterdam, Amsterdam, the Netherlands; LSU Health Sciences Center School of Dentistry, UNITED STATES

## Abstract

**Objectives:**

The composition of dental plaque has been well defined, whereas currently there is limited understanding of the composition of denture plaque and how it directly influences denture related stomatitis (DS). The aims of this study were to compare the microbiomes of denture wearers, and to understand the implications of these towards inter-kingdom and host-pathogen interactions within the oral cavity.

**Methods:**

Swab samples were obtained from 123 participants wearing either a complete or partial denture; the bacterial composition of each sample was determined using bar-coded illumina MiSeq sequencing of the bacterial hypervariable V4 region of 16S rDNA. Sequencing data processing was undertaken using QIIME, clustered in Operational Taxonomic Units (OTUs) and assigned to taxonomy. The dentures were sonicated to remove the microbial flora residing on the prosthesis, sonicate was then cultured using diagnostic colorex *Candida* media. Samples of unstimulated saliva were obtained and antimicrobial peptides (AMP) levels were measured by ELISA.

**Results:**

We have shown that dental and denture plaques are significantly distinct both in composition and diversity and that the oral microbiome composition of a denture wearer is variable and is influenced by the location within the mouth. Dentures and mucosa were predominantly made up of Bacilli and Actinobacteria. Moreover, the presence of natural teeth has a significant impact on the overall microbial composition, when compared to the fully edentulous. Furthermore, increasing levels of *Candida spp*. positively correlate with *Lactobacillus spp*. AMPs were quantified, though showed no specific correlations.

**Conclusions:**

This is the first study to provide a detailed understanding of the oral microbiome of denture wearers and has provided evidence that DS development is more complex than simply a candidal infection. Both fungal and bacterial kingdoms clearly play a role in defining the progression of DS, though we were unable to show a defined role for AMPs.

## Introduction

Healthcare improvements in the last century have led to an increasingly elderly population. Worldwide, 810 million people are aged 60 years or over, which is predicted to increase to at least two billion by 2050 (22% of the entire global population) [1]. In the EU alone the proportion of the population who are 65 years and older is predicted to reach 53% by the year 2025 [2]. This demographic change will result in significant challenges for oral healthcare delivery, to an increasingly aged population with declining oral health. As the population ages then oral diseases become more relevant with respect to their local and systemic impact, which can have profound implications for healthcare provision [3, 4].

The oral cavity is a complex environment that is continually exposed to numerous opportunistic microbial pathogens. These are kept in check by a robust arsenal of immune factors that maintain a healthy oral environment and prevent the development of disease. This arena has gradually become a key area of biomedical research, which has led to a greater understanding of the causes, pathogenesis and host response against oral disease, with the majority of research focussing on diseases affecting dentate individuals, such as gingivitis, periodontitis and caries. Conversely, there is very limited research regarding denture related disease. Despite major improvements in oral health worldwide, recent estimates report that the rate of edentulouness still varies from 7 to 69% of the worlds adult population [5, 6], and in the US and UK populations around one fifth wear some form of removable denture [7, 8]. This continued high prevalence should convince researchers that, there is a requirement to develop an understanding of the implications of dentures on oral and systemic health.

The primary condition denture wearers suffer from is denture stomatitis (DS). This refers to inflammation of the oral mucosa and pathological changes associated with the wearing of dentures [9]. DS can be classified according to the severity of inflammation using a scale first described by Newton [10, 11]. The aetiology of DS is related to a variety of factors including poorly fitting dentures causing trauma and biological factors such as poor salivary flow, smoking or antibiotic treatment as well as microbial infection [12]. However, *Candida albicans* is generally attributed as being the main causative agent in DS affecting approximately 30–70% of denture wearers [9]. *Candida spp*. colonise the denture surface, forming co-aggregates with bacteria to build complex microbial communities known as biofilms. The majority of literature in this area focuses solely on *Candida* as the primary cause of infection, however, there is increasing evidence to suggest that this is very much a polymicrobial disease in which bacterial and fungal interactions play a role in disease pathogenesis [13, 14]. Several studies have isolated bacteria directly from the surface of dentures using standard microbial culturing techniques, primarily streptococci and staphylococci species [15–18]. However, these culture based methods are largely inadequate and unlikely to give a comprehensive representation of the polymicrobial population, which can contain up to 10^11^ microbes per milligram of denture plaque [19]. The advent of high throughput sequencing however has revolutionised our understanding of microbial ecosystems, and thus using this superior method we can for the first time gain an insight into the oral microbiome of denture wearers.

In addition to an incomplete understanding of the composition of denture plaque, we also have limited knowledge and understanding of the local host immune response. It has been established that the immune response is gradually impaired with increasing age [20, 21], but with the addition of loss of natural teeth an even greater rapid decline in host protective responses in the oral cavity is reported [22]. Antimicrobial peptides (AMP) including cathelicidin LL-37, histatins and defensins, which exhibit antimicrobial and immunoregulatory properties and protect mucosal surfaces against pathogens, have been found to be present in saliva. However, no studies have yet investigated salivary AMPs in denture wearers and the potential role they may play in the protection against denture plaque. Thus, a more in depth investigation is required into the host-microbiome relationship in the denture wearing individuals, which may help towards understanding the pathogenesis of DS.

The aim of this study was therefore to carry out a detailed site specific analysis of the bacterial composition of the oral microbiome of denture wearers using high throughput 16S rRNA gene sequencing technology. Furthermore, to assess changes in the diversity and composition of the microbiome against candidal load to aid understanding of the potential bacterial-fungal interactions that may be important in DS. In addition, the relationship between host antimicrobial peptides and denture plaque composition was also investigated. This is the first study to provide such a detailed microbial analyses of denture biofilms.

## Materials and Methods

### Study participants

123 denture wearing patients attending the University of Glasgow Dental School and Hospital were enrolled in the study. Convenience sampling was used based on the patient availability on recruitment days. Patients were recruited by a designated research nurse or a PhD student. Written informed consent was obtained from all participants. Ethical approval for the study was granted by the West of Scotland Research Ethics Service (12/WS/0121). All patients wore full or partial removable dentures. A team of qualified dental clinician were responsible for the collection of samples and the recording of clinical features. Data on patient age, gender, smoking status, routine oral hygiene regimens and any history of recent antimicrobial medication were recorded on a patient questionnaire. There was no age related exclusion criteria for this study. Newton’s classification method for DS was used to score the appearance of the patient’s palatal mucosa [11]. The following scores were applied; 0 = healthy mucosa, 1 = pin-point hyperaemic lesions (localized erythema), 2 = diffuse erythema (generalized simple inflammation), and 3 = hyperplastic granular surface (inflammatory papillary hyperplasia). For standardisation, all clinicians received training to calibrate scoring the extent of erythema. Patients were excluded from the study if they were pregnant, had previous radiotherapy for the treatment of head and neck malignancy, had been receiving antimicrobial/antifungal treatment, using prescription mouthwashes or had received immunosuppressant therapy within six months previous to sampling.

### Clinical sample collection

**Fig 1:**
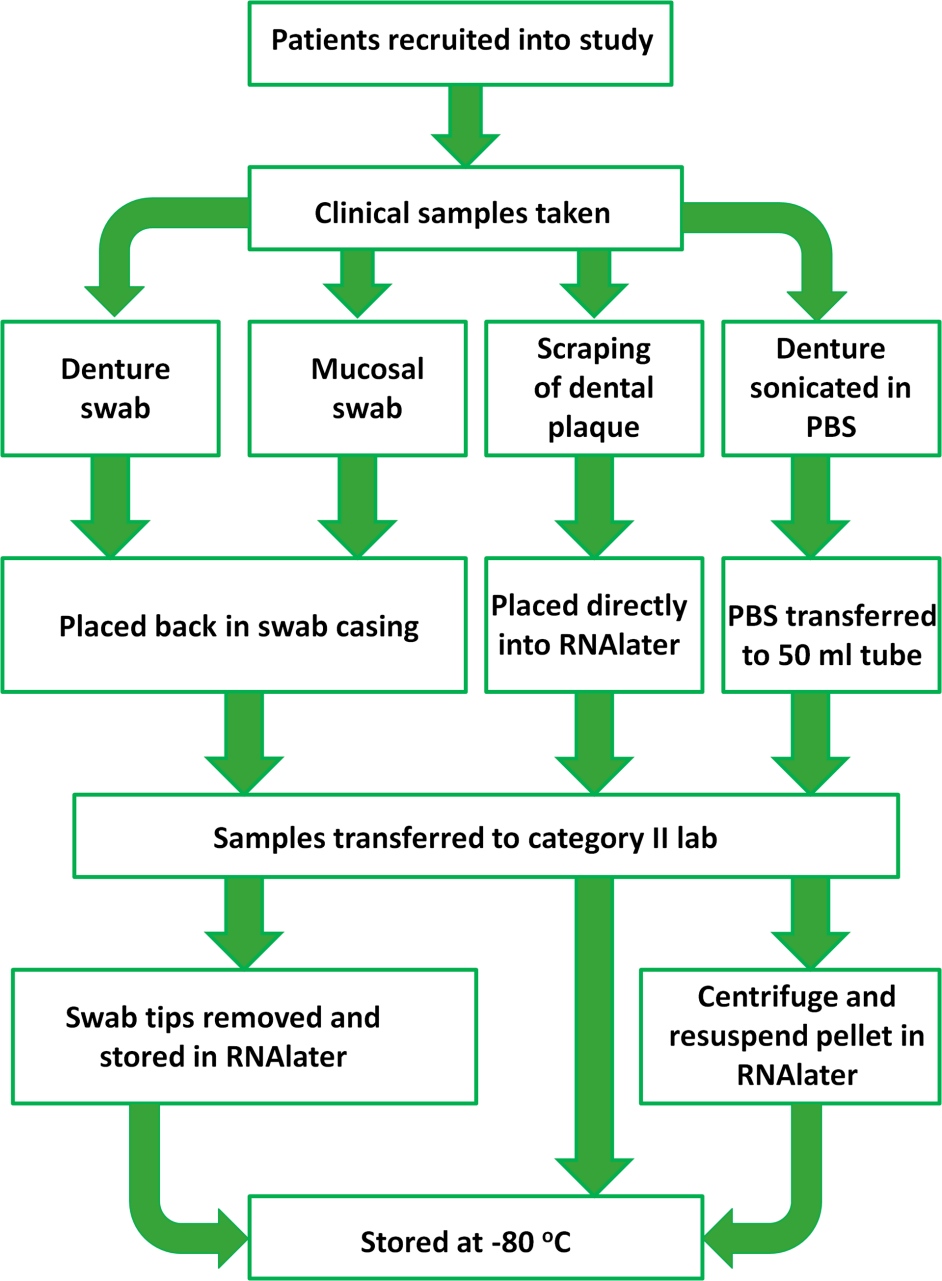
Flow chart demonstrating the process of clinical sample collection and processing in the study.

Clinical samples were collected as shown in Fig 1. Ethylene oxide sterilised swabs (Fisher Scientific, Loughborough, UK) were used to collect samples from the denture surface in contact with the palatal mucosa and the palatal mucosal surface covered by the denture. If any natural were teeth present the clinician took a supragingival plaque sample from a single tooth using a sterile dental probe, which was immediately placed into a 2 ml collection tube (Fisher Scientific) containing RNAlater^®^ (QIAgen, Manchester, UK). Dentures were removed from the patients’ mouth and placed in sterile bags (Fisher Scientific) filled with 50 ml PBS (Sigma-Aldrich, Dorset, UK), then placed in a sonic bath (Ultrawave, Cardiff, UK) for 5 min to remove adherent denture plaque. The denture sonicate was then transferred to a 50 ml tube. Denture sonicate was centrifuged for 10 min at 3700 x *g*, and the plaque pellet re-suspended in 2 ml of RNAlater (QIAgen), as previously described [23]. Swab tips were removed and stored in RNAlater. Denture plaque, dental plaque and swab samples were all stored at -80°C. In total samples from 131 patients were collected, which included 131 denture swabs, 131 mucosal swabs and 79 dental plaque samples. During DNA extraction process not all samples had sufficient DNA to use for sequencing, therefore DNA from 108 denture samples, 87 mucosal samples and 63 dental samples remained for sequencing, collectively all these samples originated from 123 patients. Samples were further separated into distinct groups for direct comparison and analysis: health and DS groups, dentate and edentate groups and complete denture and partial denture groups.

### Collection of saliva

Samples of whole unstimulated saliva were obtained by expectorating into a SaivaBio collection tube (Salimetrics, Suffolk, UK). Patients were given a maximum of 5 min to provide a sample. Saliva was clarified by centrifugation at 10,000 *g* for 10 min and then stored at -80°C.

### Candida isolation

One ml of denture sonicate was used to prepare ten-fold serial dilutions ranging from 10^0^–10^−3^. The dilutions were then used to perform colony forming unit (CFU) counts using Colorex *Candida* plates on which *C*. *albicans* colonies appeared green, *C*. *glabrata* were pink and *C*. *tropicalis* were blue. (E&O Laboratories, Bonnybridge, UK). One hundred μl of each serial dilution was spread across each plate, and then incubated at 30°C for 48 hrs. CFUs forming on plates were then counted and the average number of *Candida* cells colonising each denture was calculated.

### DNA isolation

Dental plaque samples were centrifuged for 15 min @ 13000 x *g* the supernatant was removed and the sample resuspended in 150 μl TE buffer. Swab samples were sonicated for 30 sec, and then the sonicated fluid was transferred into a deep well plate and centrifuged for 15 min at 13000 x *g*. The precipitate was then resuspended in 150 μl TE buffer. All samples were then transferred to a plate with each well containing 0.25 ml of lysis buffer (AGOWA mag Mini DNA Isolation Kit, AGOWA, Berlin, Germany), 0.3 g zirconium beads (diameter, 0.1 mm; Biospec Products, Bartlesville, OK, USA) and 0.2 ml phenol. The samples were homogenized with a Mini-beadbeater (Biospec Products) for 2 min. DNA was extracted with the AGOWA mag Mini DNA Isolation Kit.

### Quantitative PCR

Real time qPCR was performed to determine the concentration of bacterial DNA per sample as the original DNA extracted may contain DNA from other microbes such as fungi. Primers and a probe for the 16S rRNA gene were used, (F: TCCTACGGGAGGCAGCAGT, R: GGACTACCAGGGTATCTAATCCTGTT Probe: 6FAM-CGTATTACCGCGGCTGCTGGCAC-BBQ). As an internal control for PCR inhibition a qPCR of PhHV (Phocid herpesvirus type 1 gB gene) was also performed as described by Watzinger *et al* (2004) [24]. The total reaction volume was 20 μl, including 3 μl of DNA. Reactions contained a 26PCR Probe Master Mix (Roche), for 16S rRNA, 7.5 pmol primers and 3.8 pmol probe. For PhHV 1.8 pmol primers and 0.4 pmol probe of each primer was used. qPCR was carried out using the Light cycler LC480-II (Roche Diagnostics, Switzerland) under the following conditions: an activation step of 10 min at 95°C, followed by 50 cycles consisting of a denaturation step at 95°C for 30 sec, an annealing step at 60°C for 30 sec, and an extension step at 72°C for 30 sec. Bacterial 16S rDNA concentrations (CFU/ml) were determined from standard curves of *E*. *coli* K12 cultures.

### PCR amplification and Illumina sequencing

Amplicon libraries of the V4 hypervariable region of the 16S rRNA gene were generated for each of the individual samples. PCR was performed using the forward primer 515F (GTGCCAGCMGCCGCGGTAA) and the reverse primer 806R (GGACTACHVGGGTWTCTAAT) [25]. The primers included Illumina adapters and a unique 8-nt sample index sequence key [26]. The amplification mix contained 2 units of Phusion HotStart II High fidelity polymerase (Thermoscientific), 1 unit Buffer Phusion HS II [5x], including 1.5 mM MgCl_2_ (Thermoscientific), 0.2 mM dNTP (Thermoscientific, Germany) and 1 μM of each primer. To each reaction 1 ng of DNA template was added. After denaturation (98°C; 30 sec), 35 cycles of denaturation (98°C; 10 sec), annealing (55°C; 30 sec), and extension (72°C; 30 sec) were performed. Individual amplicon libraries were analyzed for DNA content with the fluorescent Quant-iT™ PicoGreen^®^ dsDNA Assay Kit (Invitrogen). The libraries were pooled in equimolar amounts. The amplicons were purified by means of the IllustraTM GFXTM PCR DNA and Gel Band Purification Kit (GE Healthcare, Eindhoven, the Netherlands). The quality and the size of the amplicons were analyzed on the Agilent 2100 (Santa Clara, CA, USA). The amplicon was sequenced in paired end mode on a MiSeq sequencing system (Illumina, Eindhoven, the Netherlands) with the v2 kit (Illumina) [26, 27].

### Sequencing data analysis

Reads were first quality filtered using Trimmomatic v0.32, [28]. Next, the reads were merged using fastq-join implemented in QIIME v.1.8.0 [29]. Sequences were clustered into operational taxonomic units (OTUs) using USEARCH v7.01090 [30], after quality filtered with usearch (maxee 0.5).The representative sequence of each cluster was assigned a taxonomy using the RDP classifier [31]. The Ribosomal Database Project: improved alignments and new tools for rRNA analysis. Nucl Acids Res 37: D141–145. doi: 10.1093/nar/gkn879) (QIIME v.1.8.0) (Greengenes v13.8 97_otus set) with a minimum confidence of 0.8.

### ELISAs

A range of antimicrobial peptides which are commonly associated with the oral cavity were selected for assessment in our patient’s saliva [32–34]. The following AMPs were assessed by ELISA, LL-37, Calprotectin, Lactoferrin, HNP1-3 (Hycult biotech, The Netherlands), Histatin 5 (Stratech scientific, Suffolk, UK) and Beta defensin 1 (BD1) (Peprotech, London, UK) as per manufacturer’s instructions. Clarified saliva samples were diluted 1:5 in assay buffer (PBS, 0.5% BSA, 0.1% Tween20). Results were calculated using a 4-parameter curve fit, quantifying colorimetric changes at 630 nm (BMG-Labtech, Ortenberg, Germany).

### Statistical analyses

The data set was randomly sub-sampled to 770 reads per sample (minimum number of reads per sample was 776) to include the maximum number of samples for analysis. OTU datasets were reduced by log2 transformation so as to carry out principal component analysis (PCA) and diversity statistics (Shannon diversity index and Dominance index); the analysis was carried out using PAST software [35].

A one-way ANOVA test was applied to compare diversity statistics at oral microbiome sites using GraphPad Prism software (version 4; La Jolla, CA, USA). PCA was used to reduce the dimensionality of the OTU dataset. 258 OTU’s were entered into the PCA. A scree plot was used to determine how many components emerged. Factor loadings above 0.15 on a component were considered to have a strong association with that component and were deemed to be the most informative in describing the microbiome components. To determine if distinct clusters formed for each group on the PCA plots, new variables were created for each principle component by using the factor loadings as regression coefficients, producing a score for each sample. These scores were then used as outcome variables to compare between groups (using t-tests where appropriate- dentate/edentate and complete/partial dentures groups).

The contribution of each bacterial class was calculated in terms of proportion to the overall sample, percentages were log transformed and a t-test was used to compare health and DS groups. Diversity statistics were compared via a t-test with GraphPad Prism v5. Spearman’s rank correlation was used to assess correlations between the abundance of individual bacterial classes or genera with the proportion of *Candida* found on dentures (CFU counts), using SPSS version 20.

Salivary concentrations of AMPs were compared between healthy and DS groups, dentate/edentate groups and complete/partial groups (S1 Table). Data were log transformed and analysed using a t-test with prism. Furthermore Spearman’s rank correlation was used to assess correlations between the abundance of individual bacterial classes or genera with the concentrations of salivary AMPs found on dentures.

To visualize the relationships and associations of the microbiomes with environmental variables canonical correspondence analysis (CCA) was applied. This form of analysis, carried out using PAST, allows the visualisation of OTU distribution and sample group distribution in relation to a number of environmental variables. Environmental variables included were *Candida* CFU counts and salivary concentration of a number of AMPs. The significance of each of the CCA axes was calculated by permuting the data 999 times.

*Candida* CFU counts were compared between healthy and DS groups, dentate/edentate groups and complete/partial groups. Data were log transformed and analysed using a t-test with GraphPad Prism v5.

### Study design

The study was designed as a pilot study and was initially only powered to detect a biologically meaningful association between diseased and healthy mouths and microbiome composition, and therefore was not originally powered to detect differences between additional variables including, denture type, dentate status, *Candida* levels and salivary AMP levels. Thus, non-significant results between these variables are not necessarily absence of effect, but a result of not achieving the full sample size required. S2 Table provides a list of the statistics carried for all the analysis.

Dentate and edentate groups were compared separately from complete and partial denture groups, as having a complete denture only applied to the upper denture from which the sample was taken. The patient however may have had natural teeth remaining on the mandible and therefore could not be classed as edentulous. The inter-correlation between complete and edentulous or partial and dentate groups are shown in Table 1. Thus given the variation in group numbers, analysis was carried out on both datasets.

**Table 1:** Patient demographics.

	Gender n (%)	Mean age	Type of Denture n (%)	Dentate status	Disease status n (%)	Mean age of denture
	*(Male)*	(years)	*(Complete upper)*	*(Edentulous)*	*(Healthy)*	(years)
	*(Female)*		*(Partial upper)*	*(Dentate)*	*(Disease)*	
Patients n = 123	43 (35)	70.7 ± 11.5 (min: 33, max: 95)	82 (67)	56 (46)	78 (63)	4.6 ± 5.3 (min:0.2, max: 40)
	80 (65)		41 (33)	67 (54)	45 (37)	

## Results

### Patient demographics

Of the 123 patients that participated in this study, 43 were male and 80 were female (Table 1). The mean (SD) patient age was 70.7 (11.5) years (min: 33, max: 95) with an average (SD) denture age of 4.6 (5.3) years (min: 0.2 max: 40). Clinical diagnoses indicated that 63% of participants had a healthy oral mucosa and the remaining 37% had an inflamed mucosa of varying severity, and were suffering from DS. The majority of patients (67%) wore complete upper dentures, while the remainder (33%) had a partial upper denture with ≥1 natural teeth remaining.

### Illumina sequencing output

Across all of the samples 632 OTUs were identified, with an average of 94 OTUs per sample (SD 39; min 25, max 254), 536 of which contained a minimum of 5 reads. The data was sub-sampled to 770 reads per sample in order to avoid bias of variable sample size. After sub-sampling 502 OTUs remained with an average of 46 OTUs per sample (SD 18; min 11, max 121). The samples were categorised into five main phyla which represented 99.6% of the reads: *Firmicutes* (40.6%), *Actinobacteria* (23%), *Bacteriodetes* (22.2%), *Proteobacteria* (9.8%) and *Fusobacteria* (4%).

### Oral microbiome by sample site

**Fig 2:**
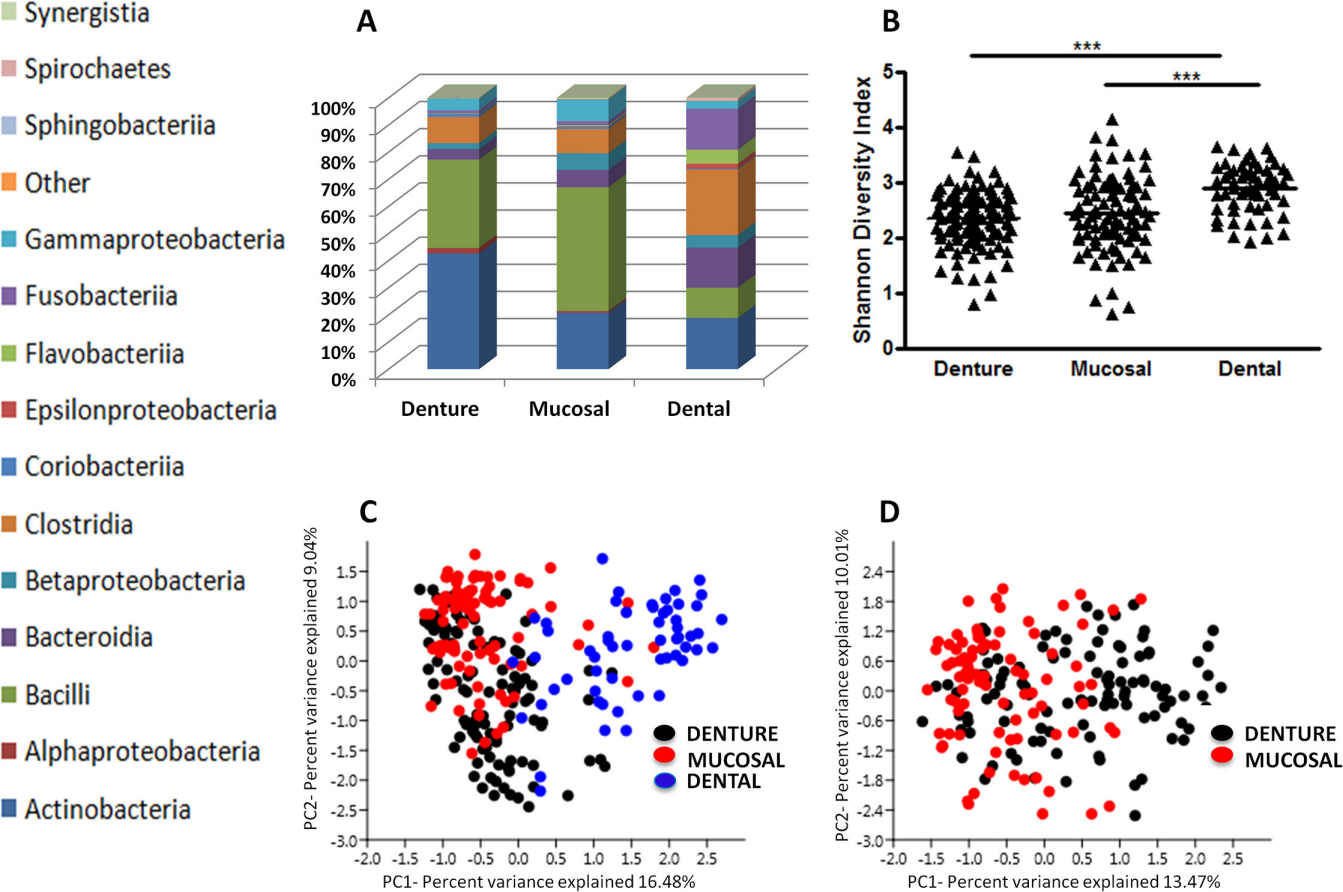
Microbial composition varies between sample sites in the oral cavity of denture wearers. One hundred and eight denture swabs, 87 mucosal swabs and 63 dental plaque samples underwent pyrosequencing of the 16S hypervariable V4 region to determine the bacterial composition of denture, palatal mucosal and dental plaque. **A)** Relative abundance of bacterial taxa at the class level per sample site. **B)** The taxonomic diversity of each group was analysed and compared via a Shannon Diversity Index. These diversity statistics show that both denture and mucosal plaque are significantly less diverse when compared to dental plaque. ***p<0.001. Principal component analysis (PCA) was applied to all sample types to reduce the multidimensionality of the data and to determine variances between **C)** Denture, Mucosal and Dental plaque. **D)** Denture and Mucosal plaque.

Samples were categorised into groups according to sample site: denture (n = 108), mucosal (n = 87) and dental (n = 63), with 337, 414 and 306 OTUs identified, respectively. On average, denture samples had 42 OTUs (SD 14; min 13, max 87), mucosal 49 OTUs (SD 20; min 11, max 121) and dental 51 OTUs (SD 37; min 16, max 306). Interestingly, denture plaque samples were found to have significantly less OTUs compared to dental, (p<0.01), and mucosal, (p<0.05), plaque. Fig 2A shows the relative abundance of bacterial taxa at the class level present in each sample type, and demonstrates considerable variation in taxonomic profiles between sites. *Actinobacteria* and *Bacilli* were the two predominant classes found at denture and mucosal sites, comprising 75.2% and 66.4%, respectively, of the overall composition when combined. However, *Actinobacteria* and *Bacilli* only contributed only 30% to dental plaque. Diversity statistics applied across samples revealed that dental plaque is more diverse (Fig 2B) than both denture plaque (Mean: 2.35 v 2.89, p<0.001), and mucosal surfaces (Mean: 2.44 v 2.89, p<0.001), with less dominant taxa according to the dominance index (p<0.001) (S1 Fig).

PCA was applied to reduce the multidimensionality of the dataset. Two primary principal components emerged from this analysis. On average, dental samples scored higher on PC1 than denture or mucosal samples (p = 0.0001) (Fig 2C), indicating higher frequencies of *Fusobacterium*, *Corynebacterium*, *Selenomonas*, *Campylobacter* and *Prevotella*, and lower of *Streptococcus* and *Rothia*. Furthermore, denture and mucosal samples were directly compared where denture samples had a higher average score on PC1 than mucosal samples (p = 0.0006) (Fig 2D). This indicated higher frequencies of *Lactobacillus*, *Actinomyces*, *Atopobium* and *Scardovia*, and lower frequencies of *Streptococcus*, *Rothia* and *Haemophilus*.

### Dentate versus edentate

**Fig 3:**
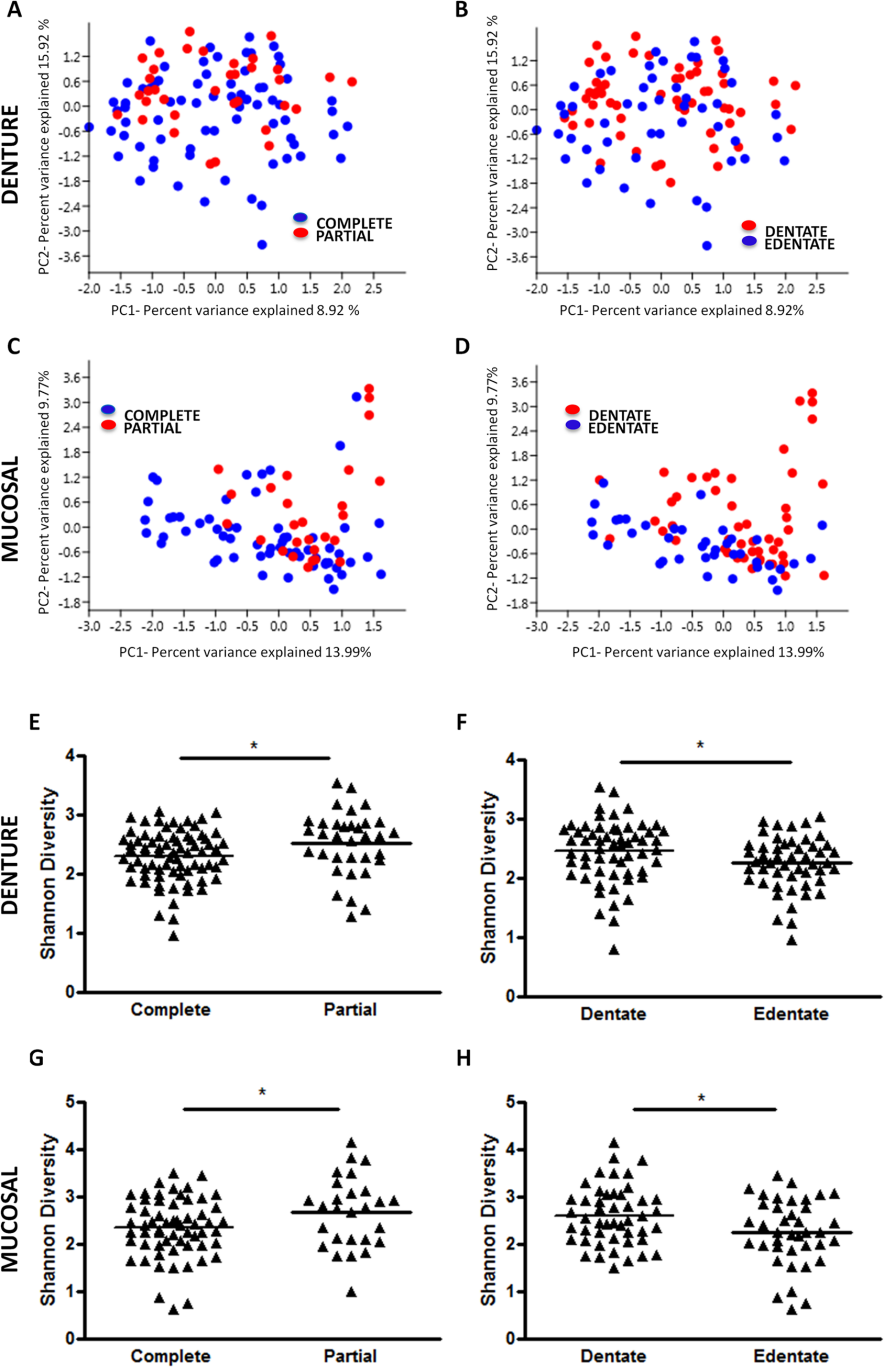
The presence of natural teeth and type of denture have significant impact of microbiome diversity. Samples were separated into groups based on denture type, (complete or partial) and dentition status (dentate or edentate) and principal component analysis was applied to the data for the denture (**A** and **B**) and mucosal microbiome (**C** and **D**) respectively. The taxonomic diversity of each group was analysed and compared via a Shannon Diversity Index for the denture (**E** and **F**) and mucosal microbiome (**G** and **H**) *p<0.05.

Samples were separated into groups depending on denture type (complete/partial) and on the presence or absence of teeth (dentate or edentate), and the microbiomes were compared at the denture and mucosal sites. PCA was applied to the data, and as previous, two principal components emerged from this analysis. When comparing denture type on the denture samples (Fig 3A), partial dentures scored higher on PC2 than complete denture samples (p = 0.0073), revealing higher frequencies of *Actinomyces*, *Haemophilus*, *Corynebacterium* and *Veillonella* and lower frequencies of *Lactobacillus* and *Streptococcus*. Denture samples were then split into dentate and edentate groups (Fig 3B). Dentate samples scored higher along PC2 than edentate (p = 0.0194), and as with the partial denture group, they had higher frequencies of *Actinomyces*, *Haemophilus*, *Corynebacterium* and *Veillonella* and lower frequencies of *Lactobacillus* and *Streptococcus*. At the mucosal microbiome partial dentures scored higher on PC1 than complete denture samples (Fig 3C) (p = 0.0001), and showed higher frequencies of *Actinomyces*, *Prevotella*, *Haemophilus* and *Neisseria* and lower frequencies of *Streptococcus*, *Lactobacillus* and *Jathinobacterium*. No differences in scores were found between dentate and edentate samples (Fig 3D).

The diversity of the same sample groups were compared and revealed that partial denture samples were significantly more diverse than those from complete dentures at both the denture, (Mean: 2.29 v 2.52, p<0.05) and mucosal surfaces, (Mean: 2.34 v 2.67, p<0.05], (Fig 3E and 3G). Furthermore, the dentate patients had a significantly more diverse microbiome than the edentate, (Fig 3F and 3H); these results were found at both the denture, (Mean: 2.45 v 2.25, p<0.05), and mucosal surfaces, (Mean: 2.6 v 2.25, p<0.05).

### Healthy versus diseased microbiome

**Fig 4:**
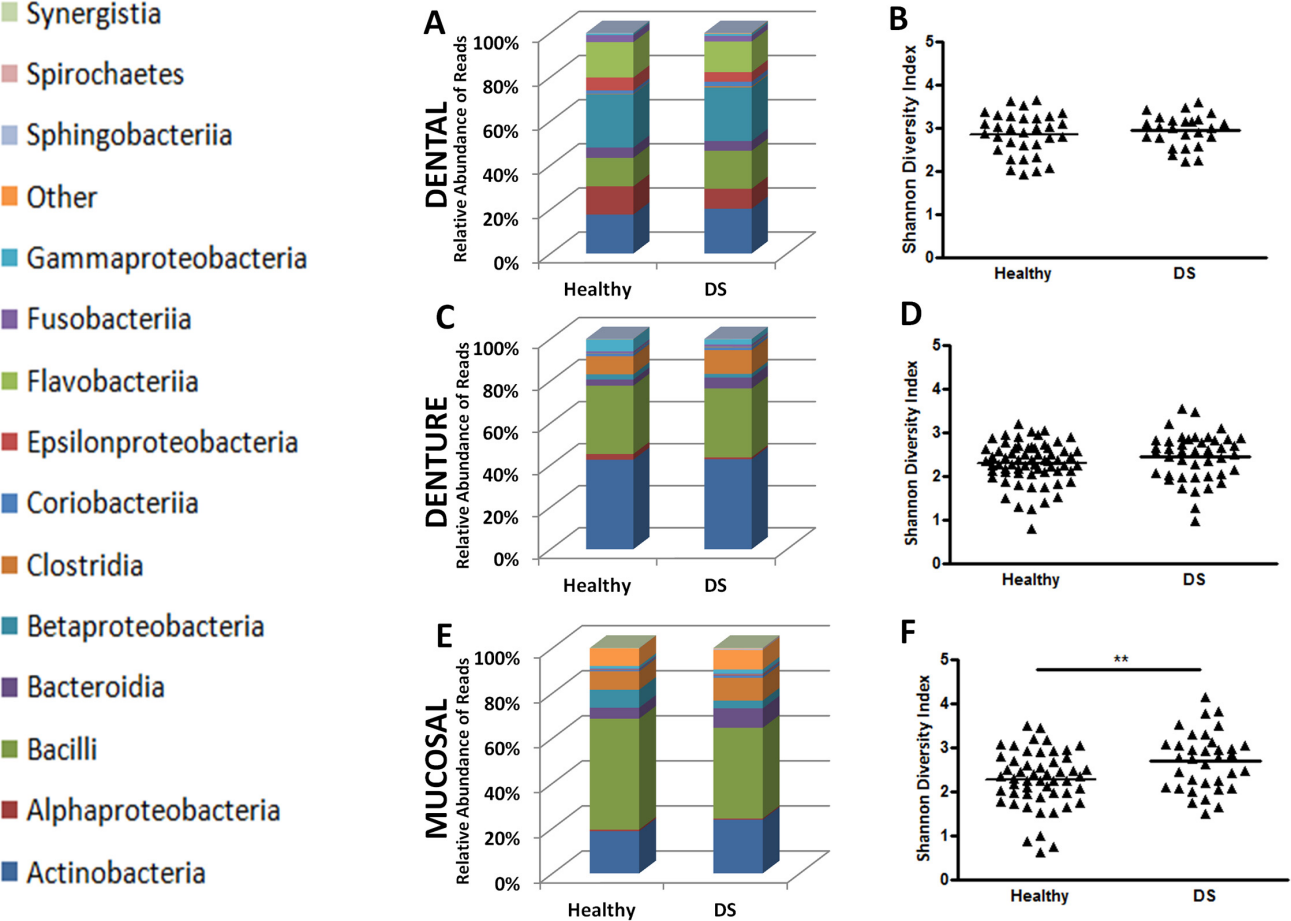
Changes in mucosal microbiome are associated with disease status. The microbiome composition was assessed for differences between health and disease in **A)** dental plaque **C)** denture plaque **E)** mucosal plaque at the class level. Differences in taxonomic diversity between health and DS were assessed. No significant differences were found in diversity in **B)** dental or **D)** denture plaque. Mucosal plaque saw significant changes in diversity as disease progressed **F)**. *p<0.05, **p<0.01.

Based on Newton’s inflammation scores, patients were separated into health and DS groups, and their microbiomes analysed. When comparing the relative abundance (%) of bacterial classes between health and DS groups, there were no differences found in dental plaque (Fig 4A). Denture plaque showed significantly higher proportion of *Bacteroidia* (Mean: 0.14 v 0.41, p<0.05), in DS patients (Fig 4C), and when investigated at the genus level, further increases in the DS group could be attributed to *Prevotella* spp. (Mean: 0.09 v 0.37, p<0.05) and *Veillonella* spp (Mean: 0.55 v 0.76, p<0.05). The mucosal microbiome showed an altered composition between health and disease, with classes *Actinobacteria* (Mean: 1.08 v 1.28, p<0.05) and *Bacteroidia* (Mean: 0.43 v 0.74, p<0.05), increasing in the latter (Fig 4E). Furthermore, Shannon diversity index statistics indicated that mucosal samples from DS patients were significantly more diverse than their healthy counterparts (Mean: 2.27 v 2.69, p<0.01) (Fig 4F), while no differences were found in dental, and denture plaque (Fig 4B and 4D). Further analysis of diversity was carried out by sub-grouping into mild inflammation (Newton’s grade 1) and severe inflammation (Newton’s grade 2&3), however, no differences that were statistically significant were observed.

### Influence of Candida on the microbiome

It was found that 72% of patient’s dentures were colonised by *Candida*. The prevalence of *Candida* species isolated from dentures of healthy and DS patients is shown in Table 2. The overall prevalence of *Candida* was higher on dentures from DS sufferers (78%) when compared with their healthy counterparts (64%). At the species level *C*. *albicans* was more predominant on DS individuals’ dentures, whereas for *C*. *glabrata* there were no differences in prevalence between health and disease. Furthermore, the number of patients in which *C*. *albicans* and *C*. *glabrata* were co-isolated was also more common in DS sufferers (36%).

**Table 2:** Prevalence of *Candida* species isolated from dentures of healthy and diseased patients.

	*Candida*+	*C*. *albicans*+	*C*. *glabrata*+	Mixed *C*. *albicans* / *C*. *glabrata*+
Healthy **n(%)** n = 78	50 (64)	32 (41)	32 (41)	14 (18)
DS **n(%)** n = 45	35 (78)	34 (76)	18(40)	16 (36)

**Fig 5:**
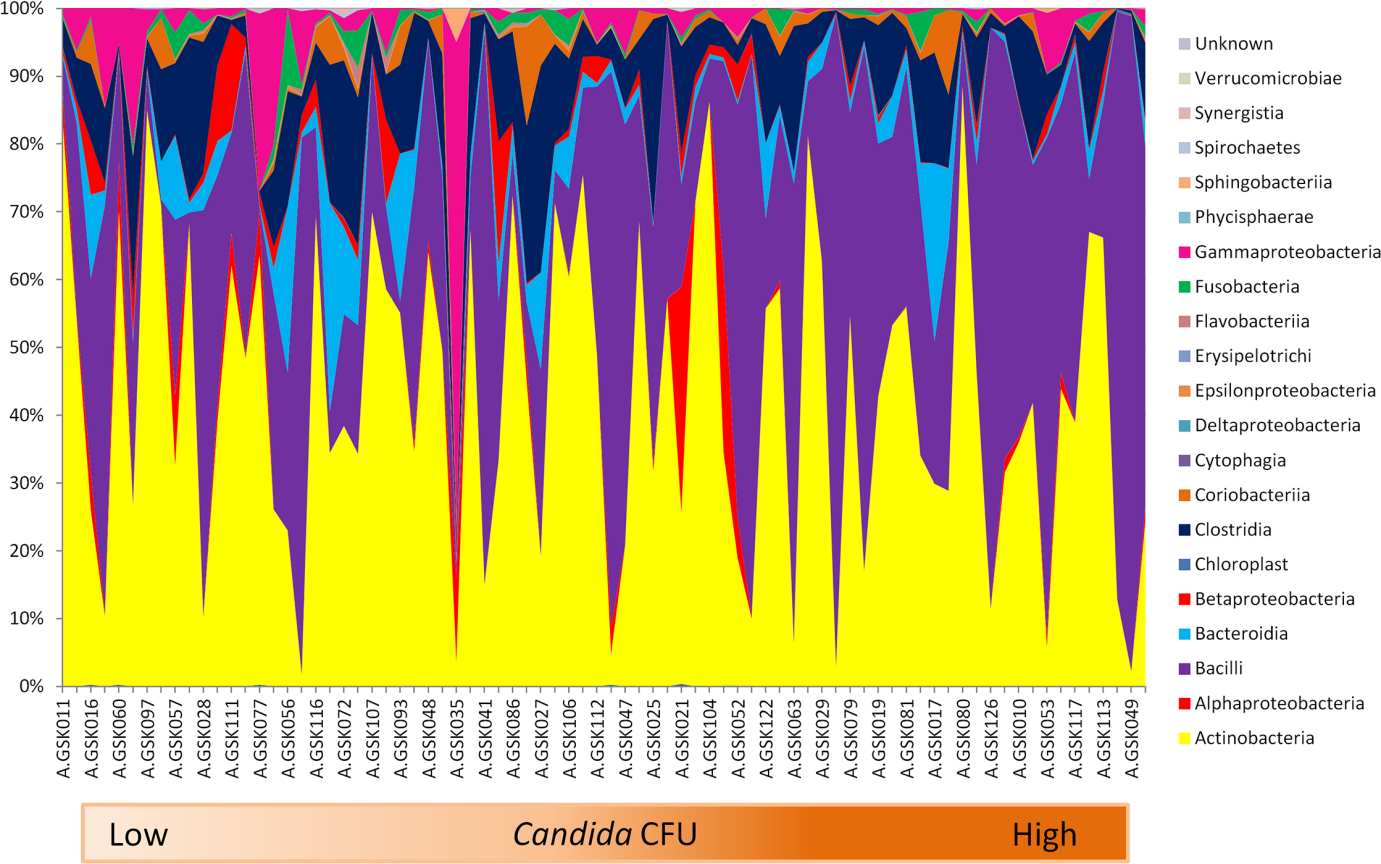
The influence of increasing *Candida spp*. CFU on the microbiome of the denture. At the denture site the relative abundance of bacterial taxa within each sample was measured against increasing *Candida* CFUs at the class level.

The influence of increasing *Candida* CFU on the bacterial microbiome of each individual was assessed at the denture and mucosal surfaces. A positive correlation was found between *Candida* CFU and *Bacilli* class on dentures (p = 0.01; Spearman’s rho 0.387), but negatively with *Fusobacteria* (p = 0.01; Spearman’s rho -0.470) (Fig 5). This was reflected at the genus level with *Lactobacillus* (p = 0.0001; Spearman’s rho 0.502) and *Fusobacteria* (p = 0.025; Spearman’s rho -0.417). No significant correlations were found between bacterial classes and *Candida* on the mucosal surface when assessing the complete cohort.

In addition to the influence of denture type and edentulism on microbiome composition and diversity, other factors were also important, including *Candida* CFU count and salivary AMP concentration. The average CFU *Candida* count found on dentures was significantly higher in individuals with a complete denture (Mean: 5.77 v 5.02, p>0.01) (S2A Fig) and in edentate individuals (Mean: 5.86 v 5.25, p<0.05) (S2B Fig), when compared to partial dentures and dentate individual respectively.

### Antimicrobial peptide detection in saliva of healthy and diseased patients

**Fig 6:**
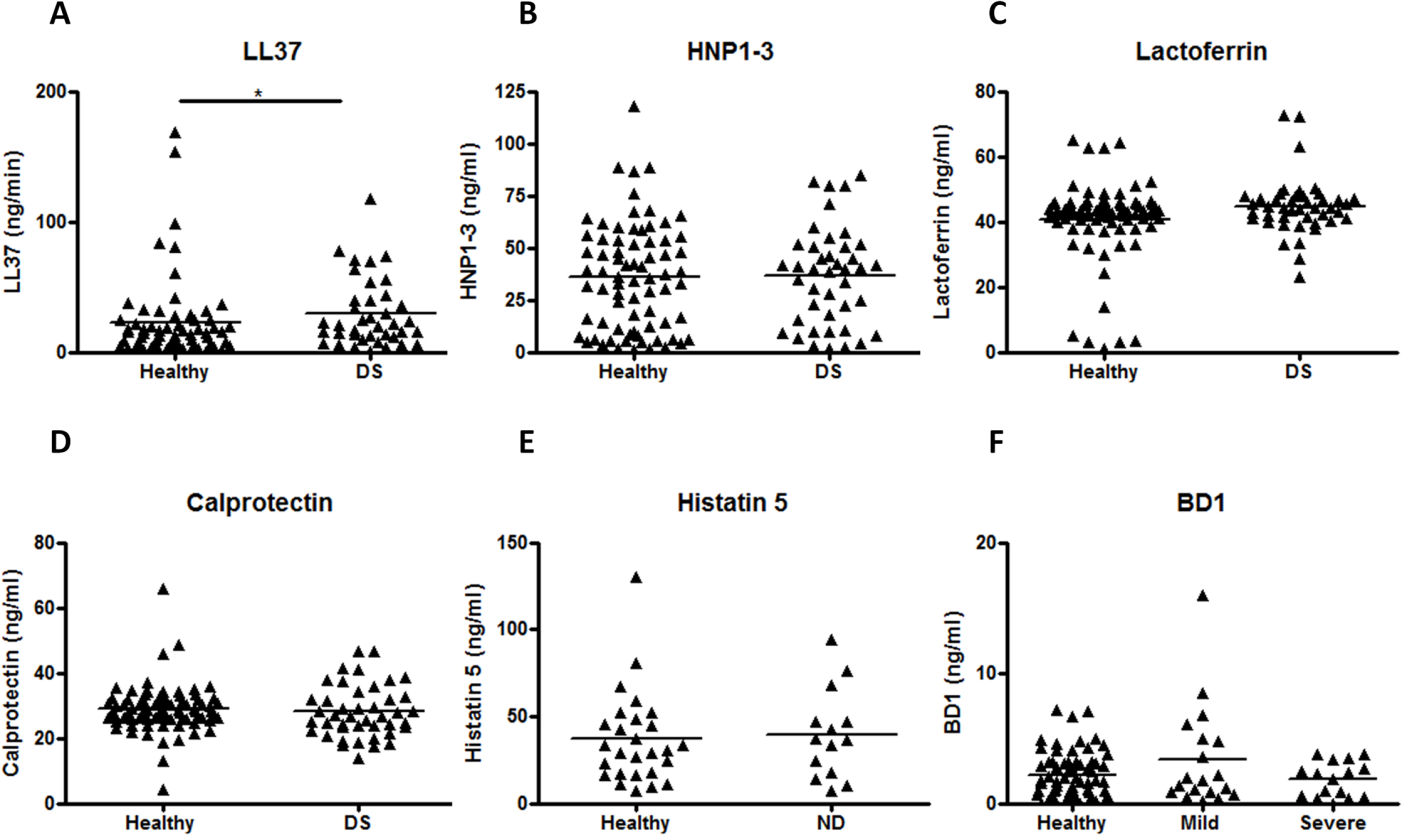
Concentrations of salivary AMPs increases with severity of inflammation. The salivary concentrations of **A)** LL37, **B)** HNP1-3, **C)** Lactoferrin, **D)** Calprotectin, **E)** Histatin 5 and **F)** BD1 were compared between healthy and diseased individuals with mild (Newton’s grade 1) or severe inflammation (Newton’s grade2&3) *p<0.05.

The levels of each AMP were compared between healthy and DS groups, and only LL-37 showed significantly elevated levels in DS patients (Mean: 1.04 v 1.28, p<0.05) (Fig 6A). HNP 1–3, lactoferrin, calprotectin, Histatin 5 and BD1, showed no statistically significant differences between groups (Fig 6B–6F). Furthermore, certain salivary AMPs, namely LL37, HNP 1–3 and Histatin 5 all showed significantly higher concentrations in patients with partial dentures (S3A–S3C Fig), and also in dentate individuals (S3G–S3I Fig). Calprotectin, Lactoferrin and BD1 all showed no differences between complete/partial (S3D–S3E Fig) or dentate/edentate groups (S3J–S3L Fig).

### Influence of salivary antimicrobial peptides on the microbiome

**Fig 7:**
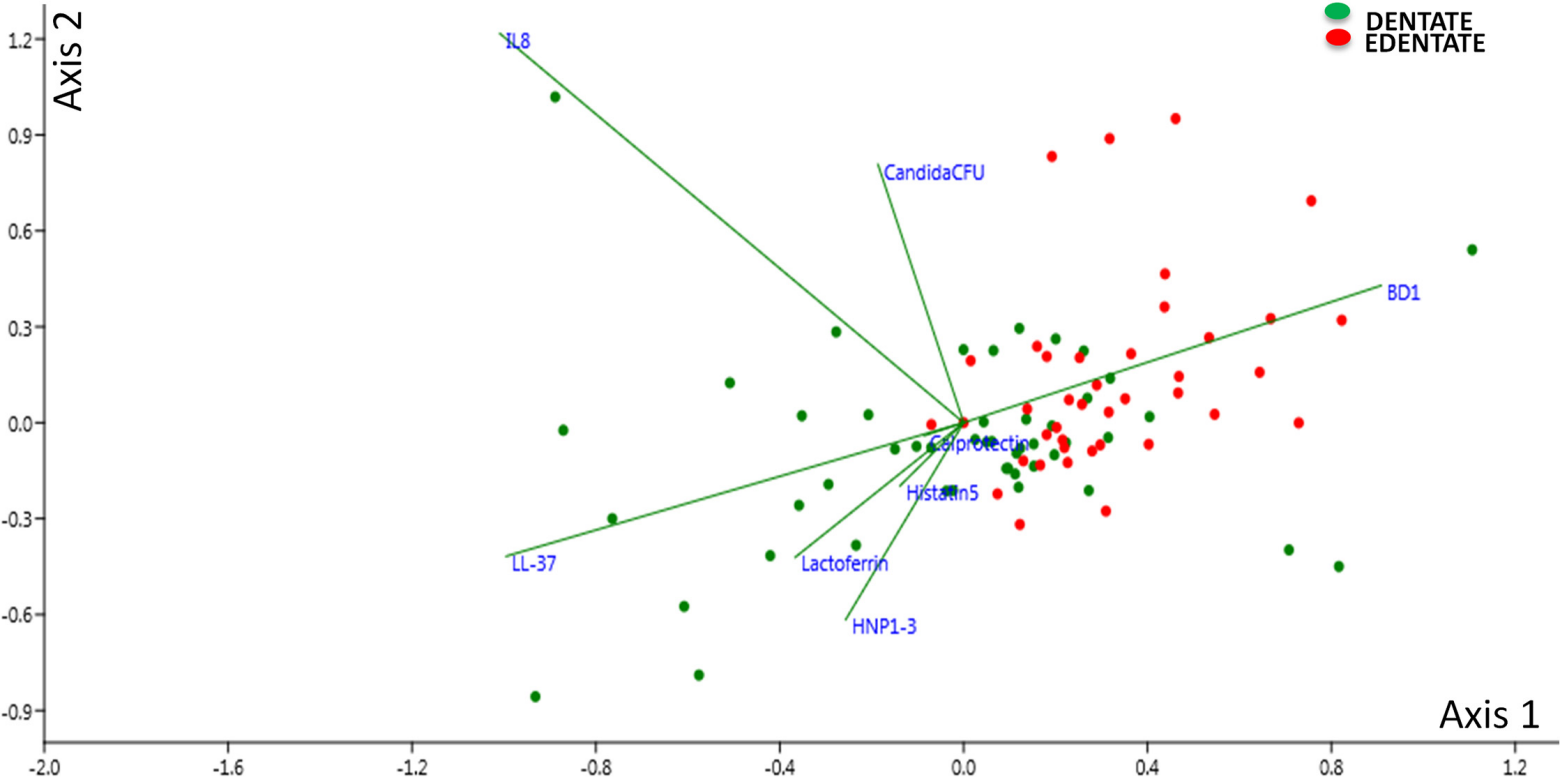
Cannonical Correspondence Analysis of the denture microbiome. CCA biplot allowing visualisation of dentate and edentate samples, represented by dots, in relation to environmental factors, including *Candida* CFU and salivary AMPs, which are represented by vectors. The variability in the samples was explained on axis 1 with 26.5% and on axis 2 with 19.3%.

Finally, we investigated the influence of the AMPs on the denture and mucosal microbiome. The relative abundance of bacterial classes and genera for the complete patient cohort was compared against increasing levels of each AMP. However, no significant AMP/ bacterial correlations were found at the denture or mucosal surface. The AMP saliva concentration was also compared against increasing *Candida* CFU, but again no significant correlations were identified. Nonetheless, CCA analysis was performed and found that at the mucosal microbiome distinct clusters formed and showed that the dentate group related stronger to the AMPs (Fig 7).

## Discussion

With the continued advancements in healthcare, the average lifespan is ever increasing, meaning that there is a larger elderly population than ever before [36]. Improving oral health has become a primary focus in medical research, and thus significant amount of time and money has been spent on investigating the pathogenesis and prevention of oral disease [6, 37]. However, this research has focused primarily on dental-related disease, whereas denture-related disease, a problem affecting a large proportion of the ever-increasing elderly population [3, 38], has been relatively understudied. Therefore, further investigation is required to elucidate its pathogenesis.

The majority of studies investigating DS focus primarily on the role of *Candida* spp. [9, 39], however given that up to 10^11^ microbes are capable of colonising the denture surface [19], then it is likely that bacteria also play a role in disease. Other studies have investigated the bacterial composition of denture plaque, however, these studies have been limited by the method of investigation, such as culturing and DNA-DNA hybridisation, which limit the number of bacterial species that can be identified [18, 40–43]. For example, Campos *et al* (2008) used PCR and identified 82 bacterial species, this however only made up approximately 50% of the bacterial flora detected in the samples, as the remainder were not-yet cultivated phylotypes and thus remained unidentified [41]. The study described herein has for the first time used detailed high-throughput 16S rRNA gene analysis of the oral microbiomes of a range of denture wearers, which gives a more comprehensive and global representation of the bacterial microbiome.

We first assessed the bacterial microbiome of three independent sites from the oral cavity of denture wearers, the denture, the palatal mucosa and dental plaque. Comparing the relative abundance of bacterial classes at the different sites showed the more diverse nature of the dental plaque in comparison to the denture and mucosal sites, both of which were predominantly composed of two classes, *Actinobacteria* and *Bacilli*. This result is unsurprising as both of these are associated with being amongst the primary colonisers within the oral cavity, particularly *Actinomyces* and *Streptococcus* species [44–46]. These results are supported by a recent study comparing biofilms forming on natural teeth against those forming on denture teeth, which demonstrated that biofilms forming on natural teeth develop quicker and have a more abundant proportion of species present [43]. PCA indicated that denture, mucosal and dental groups formed distinct clusters, thus indicating not only are they distinct in terms of diversity, but also compositionally separate. The different anatomical locations of these microbiomes likely explains these differences as the adherent surfaces and surrounding environment of dental plaque may be more suitable for the growth of a more diverse range of microorganisms [47]. For example, saliva is an important source of nutrients for microbes in the oral cavity [48], however, the presence of a denture acts as a barrier, thus with reduced nutrients available fewer organisms can form a sustainable niche.

This study has shown that the presence of natural dentition has a profound impact on the composition and diversity of the oral microbiome of a denture wearer. This suggests that this increased microbial diversity is not restricted to the anatomical sample site of the tooth, but also appears to affect the entire oral cavity. Patients included in this study that still had natural teeth remaining, ranged from 1 to 28 teeth, yet despite this wide range, PCA analysis indicated that they formed distinct groups from one another, indicating that even the presence of only one tooth is sufficient to have a profound impact of the microbiome composition. The lack of obvious groups between dentate and edentate samples at the mucosa could perhaps be explained by the nature of the surface. As we have already shown, the microbiome composition within the oral cavity is very much dependant on the location, therefore as the mucosal surface is biotic, microbes in contact are likely to be exposed to pathogen recognition receptors (PRRs) or host defence peptides from the epithelial cells [49–51]. As many of the dentate associated bacteria are not traditional commensals, they may have limited ability to colonise such a niche as well as the potential to initiate an immune response [52, 53]. Dentures, on the other hand are an abiotic surface, therefore biofilms from dentate individuals forming on the surface are likely to have less exposure to host defences and thus bacteria may grow unimpeded and have sufficient time to develop into a compositionally distinct plaque microcosm.

One of the primary aims of this study was to identify any compositional changes in the microbiome between health and disease. Denture plaque revealed a number of notable changes between health and disease, with significantly higher proportions of *Prevotella* and *Veionella* species found in denture stomatitis sufferers. This suggests that DS microbiomes have a composition more comparable to that of a dental plaque. Furthermore, at the mucosa, bacterial classes *Actinobacteria* and *Bacteroidia* also increased significantly in DS patients, further strengthening this hypothesis. A study supporting these findings by Campos *et al* (2008) investigated the microbial biofilm communities of denture stomatitis sufferers [41]. They found 32 bacterial phylotypes that were unique to denture stomatitis biofilms, a large proportion of which fell under the genera *Atopobium* (16%) and *Prevotella* (11%), both of which fall into the classes *Actinobacteria* and *Bacteroidia*, respectively. Moreover the mucosal microbiome of DS individuals is significantly more diverse than their healthy counterparts, suggesting that the compositional changes responsible for disease progression are occurring at the mucosa. However, whether these changes are cause or effect of disease is an area that merits further investigation.

We have established that the presence of natural teeth alters the composition and diversity of the microbiome, and our results suggest that the DS microbiome contains increased proportions of microbes more commonly associated with a dentate status. Therefore, we looked at the ratio of dentate to edentate within the health and DS groups and we found that both groups were equally represented within the health category (50%); however the dentate group made up a considerably larger proportion of the DS category, (61%) than the edentate (39%). Therefore, this may explain why we are seeing higher levels of these, normally dentate-associated microbes, within the microbiomes of DS individuals. Within the DS group it is likely that dentate and edentate individuals form two distinct biofilms, both with pathogenic and invasive potential, however, given that the majority of those with severe inflammation had teeth (67%), the presence of natural teeth may exacerbate denture stomatitis infection, creating a more pathogenic biofilm than those found in the edentulous.

Denture related disease is almost always attributed to infection with *C*. *albicans*, however, given the vast range of bacterial species identified on dentures and the surrounding mucosa in this study; it is unlikely that the infection can be attributed solely to *Candida* spp. Our study identified a 72% prevalence of *Candida* species on dentures, of which *C*. *albicans* was the most predominant, and was the only species in which we saw a significantly higher CFU count in DS individuals (S4A Fig). However, *C*. *glabrata* was also isolated in a high number of patients (40%), which is in line with studies showing the increasing emergence of this species, as it is currently responsible for approximately 13% of invasive candidosis cases [54–56]. In addition, the frequency of *C*. *glabrata* oral carriage rate has been shown to increase with age. Of particular interest was the number of patients in which both *C*. *albicans* and *C*. *glabrata* were co-isolated, this was high particularly amongst DS sufferers (35%). Recent studies have suggested that when co-colonised, *C*. *albicans* and *C*. *glabrata* form a more pathogenic and invasive biofilm than either species alone, thus may contribute to more severe cases of DS [57]. However, despite showing that DS is more common in dentate individuals, we found significantly higher *Candida* CFU counts on edentate patients and complete dentures when compared to dentate and partial dentures, respectively (S3 Fig). Nonetheless, this is most likely due to the less diverse microbiome of the edentulous, as with fewer microorganisms, this opens up a niche for *Candida* spp. to colonise.

Correlation analysis was carried out to investigate the affect of increasing *Candida* load (CFU counts) on the bacterial microbiome both at the class and genus level. At the denture and mucosal microbiome, there was a positive correlation with the class *Bacilli* and a negative correlation with *Fusobacteria* at the denture, these findings are in line with Kraneveld *et al* (2012), where they identified the similar correlations when analysing the effect of increasing *Candida* load on the salivary microbiome of the elderly [58]. At the genus level these correlations could be attributed to *Lactobacillus* species. This finding was surprising as the majority of literature regarding these species, indicates that they have an antagonistic relationship [59]. The mechanisms by which *Lactobacillus* inhibits growth of *Candida spp* are not fully understood. Investigations have suggested production of hydrogen peroxide by lactobacilli leads to anti-candidal activity. Furthermore lactobacilli can modulate the host response, up-regulating cytokines when co-cultured with *C*. *albicans*, which could be associated with the clearance of candidal infection. Nevertheless, denture-wearing patients have been shown to have higher levels of *Lactobacillus* species in their saliva, but they are more commonly isolated in the saliva of DS sufferers (87%) in comparison to healthy controls (65%) [60]. Yet, in spite of the vast evidence of an antagonistic interaction, certain species of oral *Lactobacillus* (namely *L*. *casei*) have demonstrated a stimulatory effect on *C*. *albicans* hyphal growth [59]. Furthermore, it has been shown that *Candida* hyphae can co-aggregate to lactobacilli and sustain their levels in patients with advanced oral diseases [60]. Nonetheless, most of these studies have focused on *Lactobacillus* spp. inhibitory effects on *C*. *albicans*, but their inhibition of other *Candida* species such as *C*. *glabrata* has proven less effective, as Jiang *et al* (2014) demonstrated that only 1 of six probiotic *Lactobacillus* species used in the study had an inhibitory effect on *C*. *glabrata* growth [61].

As well as the microbial interactions in the oral cavity of denture wearers, we were also interested in gaining a wider understanding of the host-microbial interactions. From the range of AMPs measured, only LL37 showed any significant increases between health and inflammation. This could be explained by the fact that the majority of those with inflammation (66%) were in the dentate category, whereas the healthy group were predominantly edentate (95%), and as studies have shown the concentration of salivary AMPs declines with the loss of natural teeth [22]. However, gaining a true understanding of salivary AMP concentration is more difficult with an elderly population, as the vast majority take one or more types of oral medication, many of which have been shown to cause xerostomia and thus there is the potential that they may affect salivary AMP concentrations [62]. Yet, to our knowledge the direct implication of oral medication on AMP production has not yet been elucidated.

In order to identify any potential relationships or associations between AMPs and the microbiome, correlation analysis was carried out. Each AMP was correlated with the denture and mucosal microbiome at both the class and genus level. However, no significant correlations were identified. Nonetheless, this is not to say that the AMPs are not interacting with specific microbes, it is more likely due to their broad spectrum activity [63]. Furthermore, Bals *et al* (2000) stated that establishing the individual contribution of an AMP to host defence is extremely difficult due to the complexity of the environment and host-microbial interactions [64], as we have clearly demonstrated from the data described herein.

One of the main limitations of this study is that the majority of OTUs detected have only been identified up to genus level. Therefore, this will make it more difficult to decide on the correct species to include in devising an appropriate biofilm model of DS. However, given the vast number of studies regarding the composition of the oral microbiome, there is already an excellent understanding of the bacterial species normally found in the oral cavity based on the genuses we have identified [44, 47]. Nonetheless, we still have to proceed with caution when selecting species as different species within the same genus could have drastically different physiological properties and distinct pathogenic potential.

In conclusion, given the complexity of the oral environment, comprehending the intricate interactions of this ecosystem is a difficult task, and the addition of a denture further adds to the complexity. However, this study has taken a significant step forward towards understanding this environment. With a detailed knowledge of the microbial composition, research in this area can progress further, with a more in depth focus on how these organisms interact with one another as well as the host, allowing us to develop meaningful biofilm models of denture plaque. From this study we can conclude that the bacterial microbiome composition of denture wearers is not consistent throughout the mouth and varies depending on sample site. Moreover, the diversity of dental plaque is unsurprisingly more diverse than the denture and mucosal microbiome, and with this we have shown that the presence of natural teeth has a significant impact on the overall microbial composition. Furthermore, higher levels of *Candida spp*. appear to induce a compositional switch to a more aciduric environment. Thus, we have deduced that there are a vast number of environmental variables with the potential to alter this environment, which ultimately means that the oral microbiome of each denture wearer is unique to the individual. Nonetheless, with the detailed knowledge we have gained from this study we can achieve a better understanding of the pathogenesis of denture related disease and which will aid towards the development of potential therapeutics.

**Author information:** Glasgow Dental School, School of Medicine, College of Medical, Veterinary and Life Sciences, University of Glasgow.

## Supporting Information

S1 Fig Dominance index diversity between sample sites. The taxonomic dominance of bacterial groups was analysed within each sample and compared across sample sites via a Dominance Index. Statistics indicate dental samples are more diverse than both denture and mucosal, samples. ***p<0.001.
(TIF)

S2 Fig Abundance of *Candida* on dentures is influenced by denture type and presence of natural teeth. The average CFU *Candida* counts found on dentures were compared between complete and partial **A)** and dentate and edentate **B)** patients. *p<0.05, **p<0.01.
(TIF)

S3 Fig Concentration of salivary AMPs is influenced by denture type and presence of natural teeth. The average AMP concentrations found in saliva was compared between complete and partial **A), B) C), D), E) and F)** and dentate and edentate **G), H), I), J), K) and L)** for LL37, HNP 1–3, Histatin 5, Calprotectin, Lactoferrin and BD1 respectively patients. **p<0.01, **p<0.001.
(TIF)

S4 Fig Abundance of *Candida species* on dentures. The average CFU counts of **A)** *C*. *albicans*, and **B)** *C*. *glabrata* found on dentures were compared between healthy and diseased patients. **p<0.01.
(TIF)

S1 Table Patients screened for salivary AMPs. (DOCX)

S2 Table Data Analysis Statistic. (DOCX)
