# Supplementary figures and images for: The Oral Microbiome of Denture Wearers Is Influenced by Levels of Natural Dentition
Source: PLoS One. 2015 Sep 14;10(9):e0137717. doi: 10.1371/journal.pone.0137717 (PMC4569385; doi:10.1371/journal.pone.0137717)

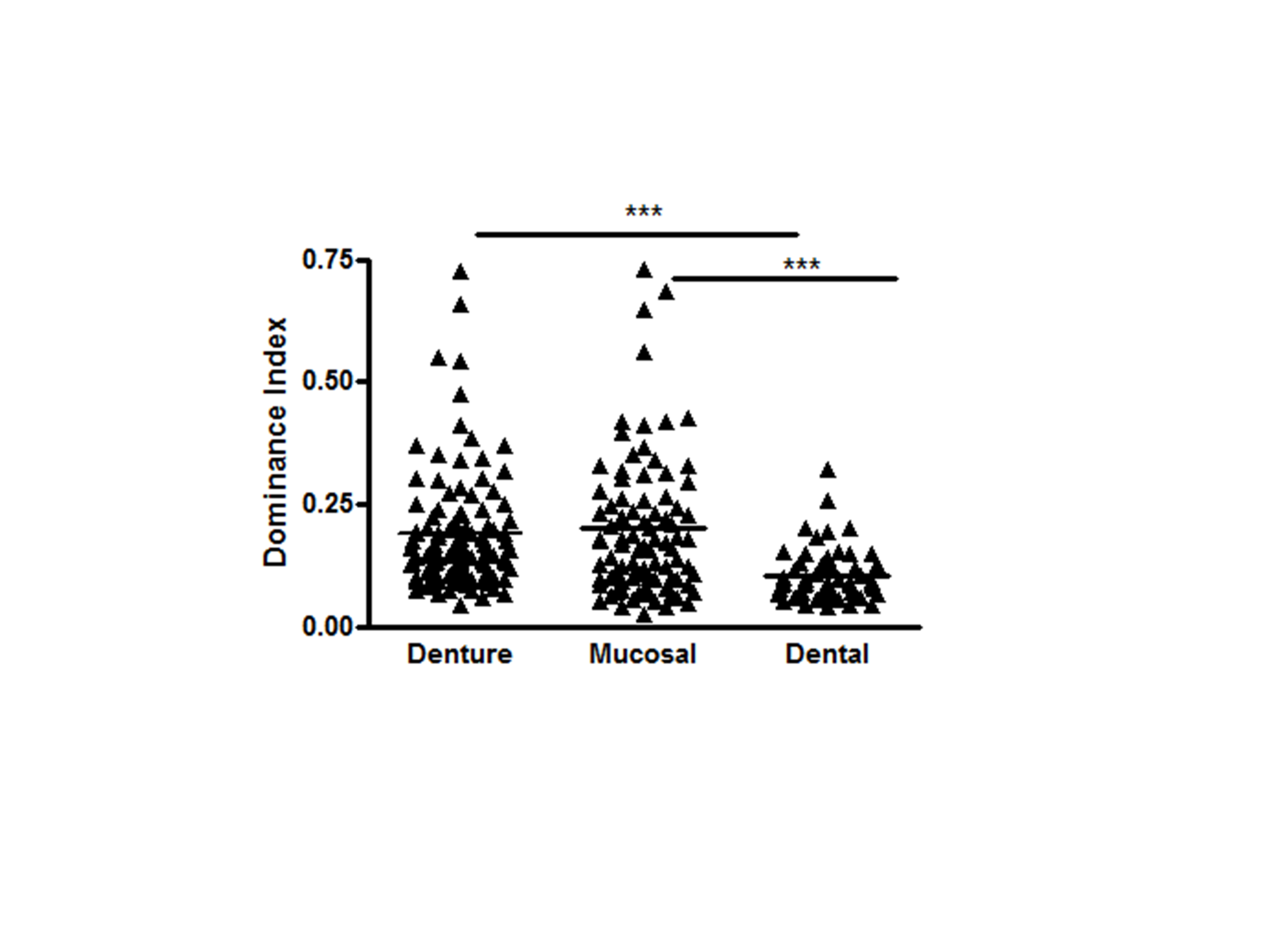

Supplement: S1 Fig — The taxonomic dominance of bacterial groups was analysed within each sample and compared across sample sites via a Dominance Index. Statistics indicate dental samples are more diverse than both denture and mucosal, samples. ***p<0.001. (TIF) [file pone.0137717.s001.tif]

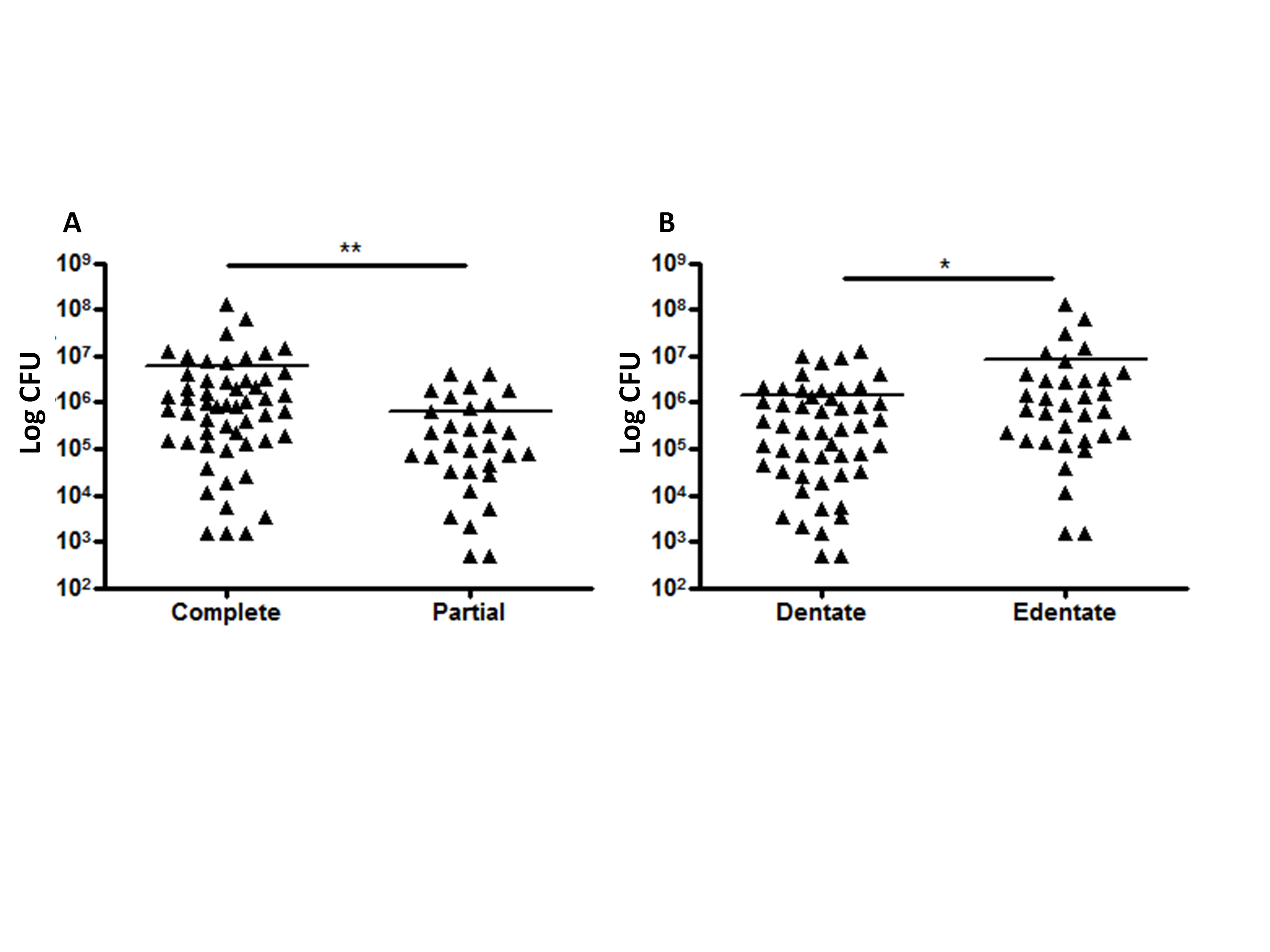

Supplement: S2 Fig — The average CFU Candida counts found on dentures were compared between complete and partial A) and dentate and edentate B) patients. *p<0.05, **p<0.01. (TIF) [file pone.0137717.s002.tif]

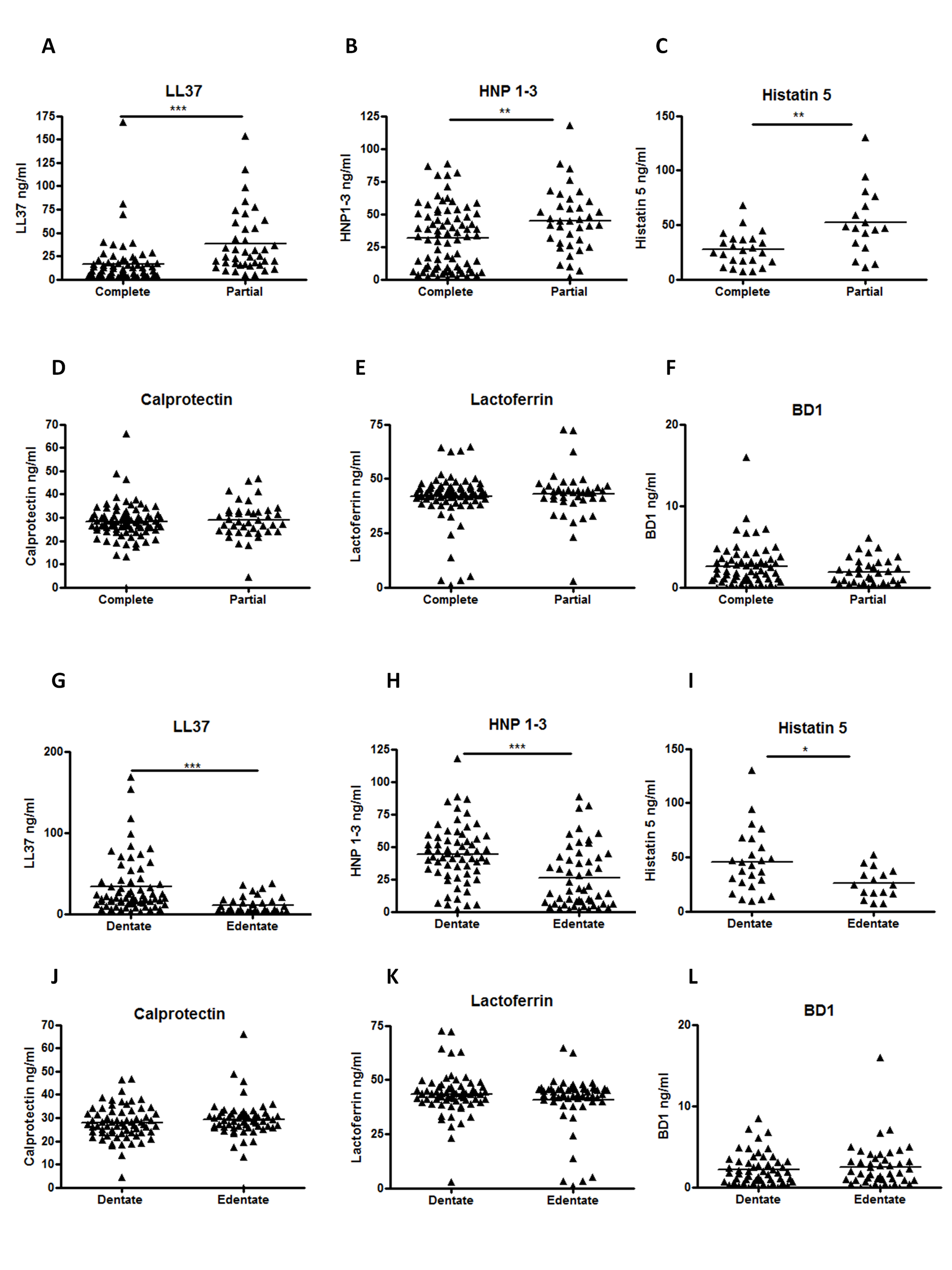

Supplement: S3 Fig — The average AMP concentrations found in saliva was compared between complete and partial A), B) C), D), E) and F) and dentate and edentate G), H), I), J), K) and L) for LL37, HNP 1–3, Histatin 5, Calprotectin, Lactoferrin and BD1 respectively patients. **p<0.01, **p<0.001. (TIF) [file pone.0137717.s003.tif]

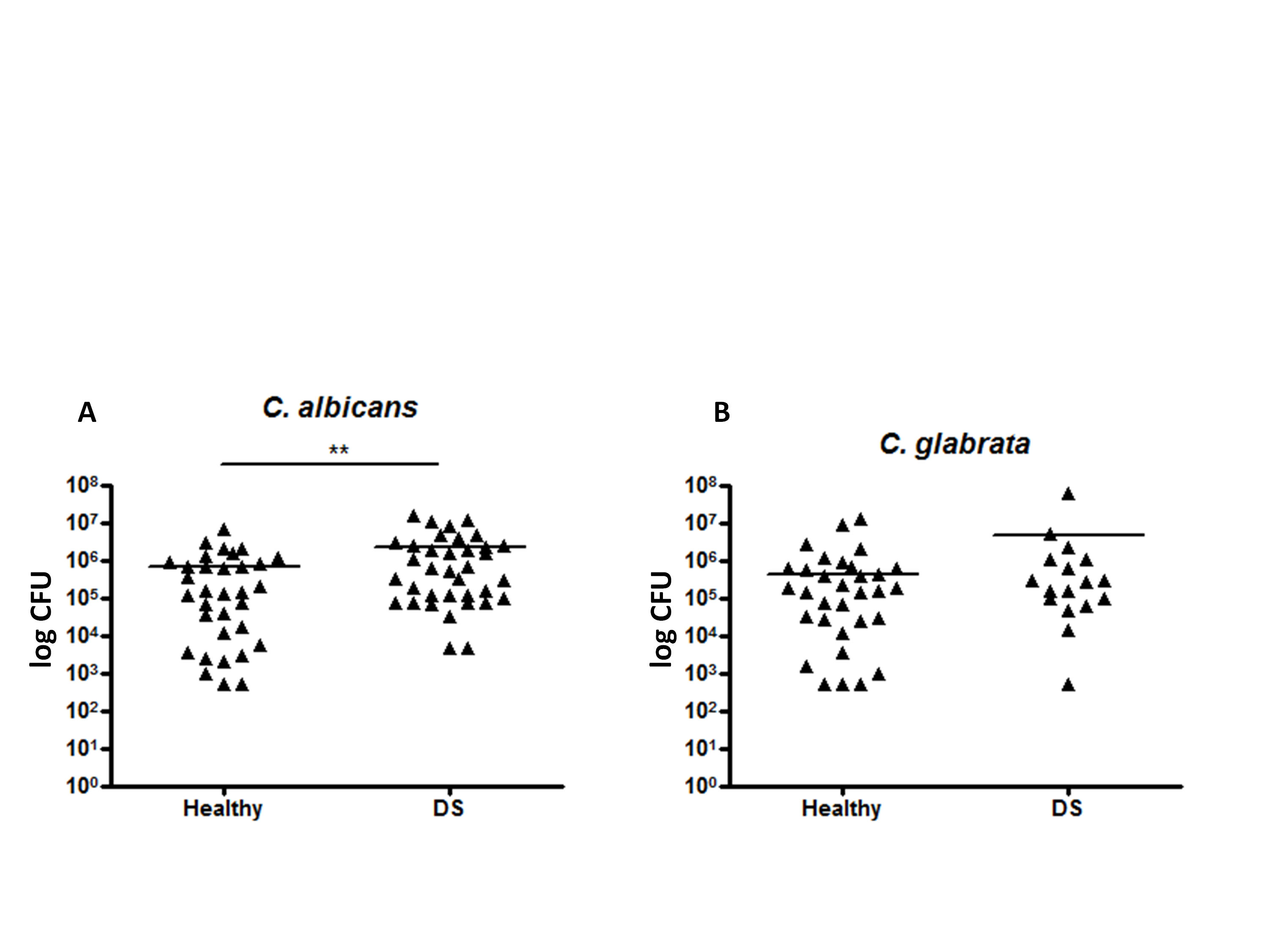

Supplement: S4 Fig — The average CFU counts of A) C. albicans, and B) C. glabrata found on dentures were compared between healthy and diseased patients. **p<0.01. (TIF) [file pone.0137717.s004.tif]
