# Supplementary material for: The Oral Microbiome of Denture Wearers Is Influenced by Levels of Natural Dentition
Source: PLoS One. 2015 Sep 14;10(9):e0137717. doi: 10.1371/journal.pone.0137717 (PMC4569385; doi:10.1371/journal.pone.0137717)
Supplement: S1 Table — (DOCX) [file pone.0137717.s005.docx]

| **AMP (n)** | **Healthy**  **n(%)** | **DS**  **n(%)** | **Dentate**  **n(%)** | **Edentulous**  **n(%)** | **Partial**  **n(%)** | **Complete**  **n(%)** |
| --- | --- | --- | --- | --- | --- | --- |
| **LL37 (103)** | 64 (62) | 39 (38) | 66 (64) | 37 (36) | 63 (61) | 40 (39) |
| **Lactoferrin (125)** | 81 (65) | 44 (35) | 68 (55) | 57 (45) | 40 (32) | 85 (68) |
| **Calprotectin (124)** | 81 (65) | 43 (35) | 67 (54) | 57 ((46) | 41 (33) | 83 (67) |
| **HNP 1-3 (116)** | 74 (64) | 42 (36) | 63 (54) | 53 (46) | 79 (68) | 37 (32) |
| **BD1 (100)** | 65 (65) | 35 (35) | 54 (54) | 46 (46) | 65 (65) | 35 (35) |
| **Histatin 5 (40)** | 27 (68) | 13 (32) | 25 (63) | 15 (37) | 23 (58) | 17 (42) |
