# Supplementary material for: The Oral Microbiome of Denture Wearers Is Influenced by Levels of Natural Dentition
Source: PLoS One. 2015 Sep 14;10(9):e0137717. doi: 10.1371/journal.pone.0137717 (PMC4569385; doi:10.1371/journal.pone.0137717)
Supplement: S2 Table — (DOCX) [file pone.0137717.s006.docx]

| **Fig** | **Difference of Means** | **Confidence Interval (95%)** | **Significance (p)** |
| --- | --- | --- | --- |
| B (Denture v Mucosal)  2B (Denture v Dental)  2B (Mucosal v Dental) | 0.086  0.536  0.449 | -0.2794 to 0.1076  -0.7554 to -0.3156  0.6785 to -0.2207 | p>0.05  p< 0.01  p< 0.01 |
| 2C (PC1) | 10.99 | -12.12 to -9.853 | p< 0.0001 |
| 2D (PC1) | 4.165 | -5.400 to -2.930 | p< 0.0001 |
| 3A (PC1)  (PC2) | 0.2684  2.308 | -2.047 to 2.584  -3.983 to -0.6328 | p>0.05  p< 0.01 |
| 3B (PC1)  (PC2) | 0.4587  1.877 | -2.611 to 1.694  -3.447 to -0.3072 | p>0.05  p< 0.05 |
| 3C (PC1)  (PC2) | 4.725  0.0396 | -7.001 to -2.450  -2.067 to 2.146 | p< 0.0001  p>0.05 |
| 3D (PC1)  (PC2) | 1.996  0.337 | -4.264 to 0.2731  -1.606 to 2.280 | p>0.05  p>0.05 |
| 3E | 0.2218 | -0.4158 to -0.02782 | p< 0.05 |
| 3F | 0.1996 | 0.01073 to 0.3885 | p< 0.05 |
| 3G | 0.338 | -0.6444 to -0.03157 | p< 0.05 |
| 3H | 0.3505 | 0.06739 to 0.6336 | p< 0.05 |
| 4B | 0.0835 | -0.3205 to 0.1535 | p>0.05 |
| 4D | 0.1392 | -0.3337 to 0.05537 | p>0.05 |
| 4F | 0.4143 | -0.6978 to -0.1309 | p< 0.01 |
| 6A | 0.2409 | -0.4567 to -0.02501 | p< 0.05 |
| 6B | 0.03908 | -0.2165 to 0.1383 | p>0.05 |
| 6C | 0.08815 | -0.1810 to 0.004677 | p>0.05 |
| 6D | 0.01308 | -0.03338 to 0.05955 | p>0.05 |
| 6E | 0.02414 | -0.2304 to 0.1821 | p>0.05 |
| 6F | 0.4837 | -1.450 to 0.4823 | p>0.05 |
| S1 (Denture v Mucosal)  (Denture v Dental)  (Mucosal v Dental) | 0.00956  0.08651  0.09607 | -0.05081 to 0.03169  0.03964 to 0.1334  0.04728 to 0.1449 | P > 0.05  P < 0.001  P < 0.001 |
| S2A | 0.7486 | 0.2482 to 1.249 | p< 0.01 |
| S2B | 0.6086 | -1.108 to -0.1087 | p< 0.05 |
| S3A | 0.5130 | -0.7082 to -0.3178 | p<0.0001 |
| S3B | 0.2543 | -0.4312 to -0.07743 | p< 0.01 |
| S3C | 0.2747 | -0.4523 to -0.09709 | p< 0.01 |
| S3D | 0.0079 | -0.03948 to 0.05519 | p>0.05 |
| S3E | 0.02918 | -0.1248 to 0.06642 | p>0.05 |
| S3F | 0.1997 | 0.02657 to 0.3729 | p>0.05 |
| S3G | 0.5775 | 0.3868 to 0.7681 | p<0.0001 |
| Supp 3H | 0.3708 | 0.2140 to 0.5277 | p<0.0001 |
| **Figure** | **Difference of Means** | **Confidence Interval (95%)** | **Significance (p)** |
| Supp 3I | 0.2360 | 0.04826 to 0.4237 | p<0.05 |
| Supp 3J | 0.0340 | -0.07814 to 0.01013 | p>0.05 |
| Supp 3K | 0.0738 | -0.01546 to 0.1631 | p>0.05 |
| Supp 3L | 0.0718 | -0.2455 to 0.1019 | p>0.05 |
| Supp 4A | 1.699 | -2.866 to -0.5318 | p<0.01 |
| Supp 4B | 1.289 | -2.958 to 0.3798 | p>0.05 |
